# Biological characterization of coronavirus noncanonical transcripts in vitro and in vivo

**DOI:** 10.1186/s12985-023-02201-0

**Published:** 2023-10-12

**Authors:** Ching-Hung Lin, BoJia Chen, Day-Yu Chao, Feng-Cheng Hsieh, Chien-Chen Lai, Wei-Chen Wang, Cheng-Yu Kuo, Chun-Chun Yang, Hsuan-Wei Hsu, Hon-Man-Herman Tam, Hung-Yi Wu

**Affiliations:** 1 Graduate Institute of Veterinary Pathobiology, College of Veterinary Medicine, National Chung Hsing University, Taichung, 40227 Taiwan; 2 Graduate Institute of Microbiology and Public Health, College of Veterinary Medicine, National Chung Hsing University, Taichung, 40227 Taiwan; 3 Institute of Molecular Biology, College of Life Sciences, National Chung Hsing University, Taichung, 40227 Taiwan; 4 Doctoral Program in Microbial Genomics, National Chung Hsing University and Academia Sinica, Taichung, 40227 Taiwan; 5 Department of Post-Baccalaureate Medicine, College of Medicine, National Chung Hsing University, Taichung, 40227 Taiwan

**Keywords:** Coronavirus, Noncanonical transcript, Pathogenesis, Gene expression, Coronavirus genome

## Abstract

**Background:**

In addition to the well-known coronavirus genomes and subgenomic mRNAs, the existence of other coronavirus RNA species, which are collectively referred to as noncanonical transcripts, has been suggested; however, their biological characteristics have not yet been experimentally validated in vitro and in vivo.

**Methods:**

To comprehensively determine the amounts, species and structures of noncanonical transcripts for bovine coronavirus in HRT-18 cells and mouse hepatitis virus A59, a mouse coronavirus, in mouse L cells and mice, nanopore direct RNA sequencing was employed. To experimentally validate the synthesis of noncanonical transcripts under regular infection, Northern blotting was performed. Both Northern blotting and nanopore direct RNA sequencing were also applied to examine the reproducibility of noncanonical transcripts. In addition, Northern blotting was also employed to determine the regulatory features of noncanonical transcripts under different infection conditions, including different cells, multiplicities of infection (MOIs) and coronavirus strains.

**Results:**

In the current study, we (i) experimentally determined that coronavirus noncanonical transcripts were abundantly synthesized, (ii) classified the noncanonical transcripts into seven populations based on their structures and potential synthesis mechanisms, (iii) showed that the species and amounts of the noncanonical transcripts were reproducible during regular infection but regulated in altered infection environments, (iv) revealed that coronaviruses may employ various mechanisms to synthesize noncanonical transcripts, and (v) found that the biological characteristics of coronavirus noncanonical transcripts were similar between in vitro and in vivo conditions.

**Conclusions:**

The biological characteristics of noncanonical coronavirus transcripts were experimentally validated for the first time. The identified features of noncanonical transcripts in terms of abundance, reproducibility and variety extend the current model for coronavirus gene expression. The capability of coronaviruses to regulate the species and amounts of noncanonical transcripts may contribute to the pathogenesis of coronaviruses during infection, posing potential challenges in disease control. Thus, the biology of noncanonical transcripts both in vitro and in vivo revealed here can provide a database for biological research, contributing to the development of antiviral strategies.

**Supplementary Information:**

The online version contains supplementary material available at 10.1186/s12985-023-02201-0.

## Background

Coronaviruses (CoVs), which belong to the family *Coronaviridae*, order *Nidovirales*, are common contagious pathogens of humans and animals, causing widespread and costly diseases, including COVID-19 caused by severe acute respiratory syndrome coronavirus 2 (SARS-CoV-2) [1, 2]. CoVs are single-stranded, positive-sense RNA viruses with the largest known viral RNA genomes, ~ 30 kilobases (kb) [3, 4]. The genome consists of a cap, a 5′ untranslated region (UTR), open reading frames (ORFs), a 3′ UTR and a 3′ poly(A) tail. The 5′ two-thirds of the genome contains two ORFs (ORF 1a and ORF 1b) that encode 15–16 nonstructural proteins (nsps), and the other one-third of the genome consists largely of genes encoding structural and accessory proteins [5].

During coronavirus infection, in addition to synthesis of the coronavirus genome (referred to as coronavirus replication), a 3′-coterminal nested set of subgenomic mRNAs (sgmRNAs) are also produced (in referred to as coronavirus transcription), from which structural and accessory proteins are translated [6]. Synthesis of the sgmRNA in coronaviruses requires a discontinuous step guided by a conserved transcription regulatory sequence (TRS) motif, which is located immediately downstream of the leader sequence (TRS-L) and upstream of each structural and accessory protein-encoding gene (TRS-B) [5]. TRS-L, which is located at the 5′ terminus of the coronavirus genome, shares high sequence identity with TRS-B. TRS-L acts as an acceptor for the complementary TRS-B donor sequence during sgmRNA synthesis [5] through a similarity-assisted copy-choice mechanism. Furthermore, it has also been demonstrated that, in addition to the genome, longer sgmRNAs can also serve as templates for the synthesis of shorter sgmRNAs, extending the coronavirus transcription mechanism [7]. A subsequent study experimentally identified the leaderless genome and sgmRNAs in bovine coronavirus (BCoV) [8]. These results indicated that there are unidentified sgmRNAs with various structures in infected cells.

The defective viral genome (DVG) is a mini version of the viral genome because it consists of a deleted virus genome synthesized presumably through a recombination process. DVG has been identified in most RNA viruses during infection [9–11]. In coronaviruses, few DVG (or defective interfering RNA) species have been experimentally identified, including bovine coronavirus (BCoV) and mouse hepatitis viruses (MHVs) [12]. Because these previously identified DVGs in coronaviruses contain *cis*-acting elements required for gene expression in their 5′ and 3′ termini, they are employed as surrogates for the ~ 30 kb full-length genome in studies on coronavirus replication, transcription and translation [7, 13–19].

In addition to the well-known coronavirus genomes and sgmRNAs described above, other coronavirus RNA species, which are collectively referred to as noncanonical transcripts, have been suggested. However, their biological features have not yet been experimentally characterized in cell cultures and animals. In the current study, with the assistance of nanopore direct RNA sequencing, the synthesis of coronavirus noncanonical transcripts was experimentally validated, and the biological features were characterized in cell cultures and animals. The identification of the biological characteristics of coronavirus noncanonical transcripts may assist the coronavirus research community in obtaining insights into coronavirus gene expression and pathogenesis and provide a database for a variety of biomedical studies.

## Methods

### Viruses, cells and animals

The Mebus strains of BCoV (GenBank: U00735.2) and MHV-A59 (GenBank: NC_048217.1) were obtained from David A. Brian (University of Tennessee, TN). Human rectum tumor (HRT)-18 cells and mouse L (ML) cells were also obtained from David A. Brian (University of Tennessee, TN). BCoV was grown in HRT-18 cells [20], and MHV-A59 was grown in ML cells. Both viruses were plaque-purified. Human embryonic kidney (HEK)-293T cells were obtained from Wei-Li Hsu (National Chung Hsing University, Taiwan). The aforementioned cells were grown in Dulbecco’s modified Eagle’s medium (DMEM) supplemented with 10% fetal bovine serum (HyClone, UT, USA) at 37 °C with 5% CO_2_ as previously described [21, 22]. Mice were maintained according to the guidelines established in the “Guide for the Care and Use of Laboratory Animals” prepared by the Committee for the Care and Use of Laboratory Animals of the Institute of Laboratory Animal Resources Commission on Life Sciences, National Research Council, USA. The animal study was reviewed and approved (IACUC No.: 108-110) by the Institutional Animal Care and Use Committee of National Chung Hsing University, Taiwan.

### RNA preparation for nanopore direct RNA sequencing

For nanopore direct RNA sequencing in vitro, HRT-18 and ML cells were infected with BCoV and MHV-A59 at a multiplicity of infection (MOI) of 0.1, and total cellular RNA was collected at 24 and 20 h postinfection, respectively. For nanopore direct RNA sequencing in vivo, 3-week-old male and specific pathogen-free BALB/c mice (BioLASCO Taiwan Co., Ltd.) were infected by intraperitoneal inoculation of 10^6^ PFU of MHV-A59 in 500 µl of DMEM. The livers of MHV-A59-infected mice were collected at 3 days postinfection. TRIzol (Thermo Fisher Scientific, Waltham, USA) was used to collect total cellular RNA from MHV-A59-infected cells and MHV-A59-infected livers according to the manufacturer’s instructions. Poly(A)-tailed RNA used for nanopore direct RNA sequencing was obtained using oligo d(T)25 magnetic beads (New England Biolabs, USA) according to the manufacturer’s instructions.

### Nanopore direct RNA sequencing

For nanopore direct RNA sequencing, 500 ng of poly(A)-containing RNA was used for library preparation with yeast enolase II (YHR174W) mRNA as a calibration standard according to the manufacturer’s instructions (MinION RNA sequencing kit, Oxford Nanopore Technologies). The prepared library was loaded onto an ONT FLO-MIN106D flow cell, and sequencing was conducted for 24 h on a MinION device (Oxford Nanopore Technologies).

### Data analyses for nanopore direct RNA sequencing

**Fig. 1:**
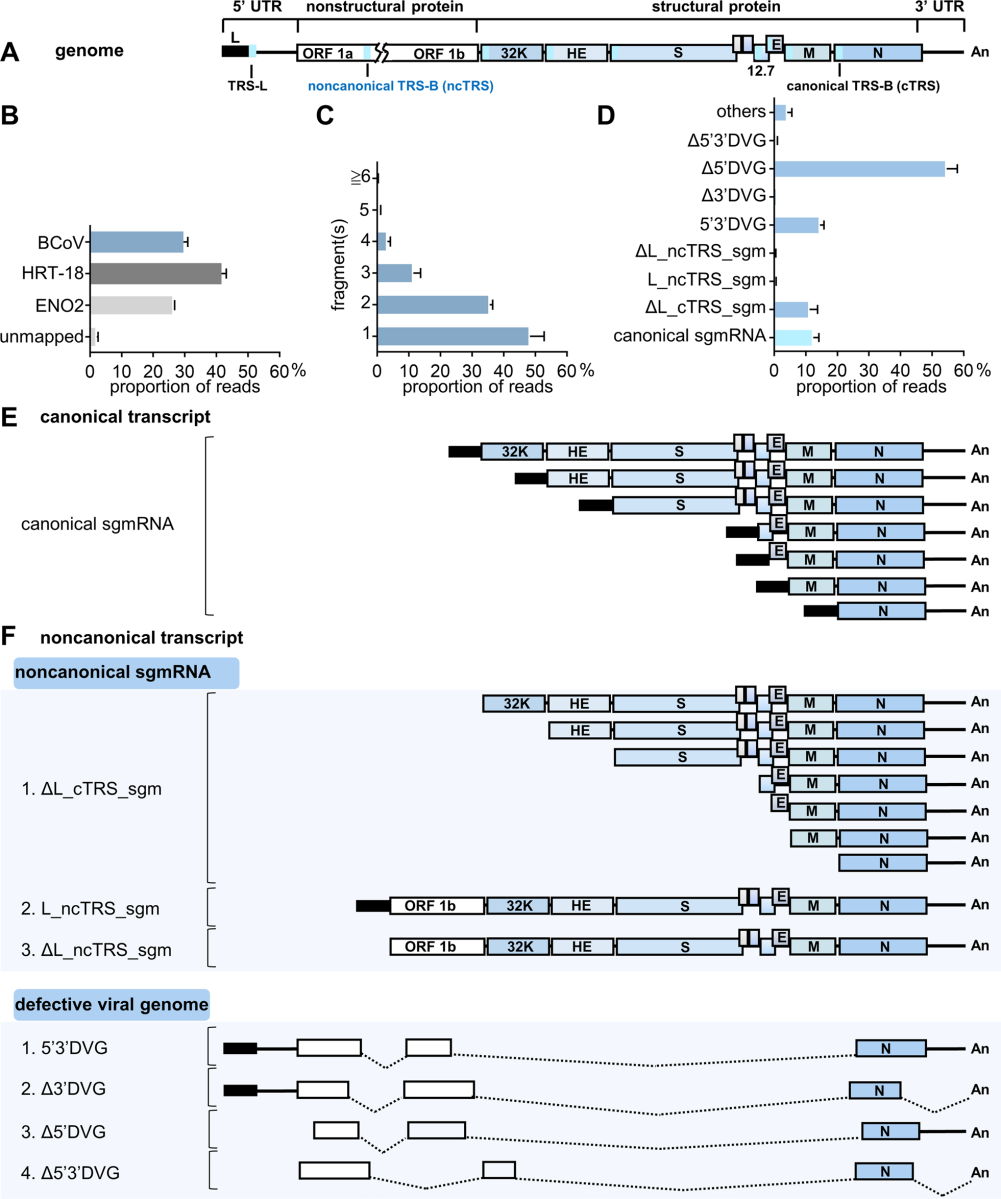
The amounts and structures of noncanonical coronavirus transcripts in BCoV-infected HRT-18 cells based on nanopore direct RNA sequencing. **A** Schematic diagram of the BCoV genome structure. TRSs, including TRS-L, noncanonical TRS-B (ncTRS) and canonical TRS-B (cTRS), are denoted by solid blue rectangles. L, leader. **B** The amounts of BCoV transcripts in BCoV-infected HRT-18 cells based on the read counts of nanopore direct RNA sequencing. ENO2, yeast enolase II mRNA. **C** The amounts of BCoV transcripts based on the number of fragments of which the transcripts are composed. **D** The amounts of each classified BCoV transcript. **E** Schematic diagram showing the structures of canonical BCoV transcripts (i.e., canonical sgmRNAs). **F** Schematic diagram illustrating the structures of noncanonical BCoV transcripts, which are divided into 2 main populations: noncanonical sgmRNAs and defective viral genomes (DVGs) (see text for details). The data in (**B**–**D**) represent the means of two independent experiments. UTR, untranslated region; ORF, open reading frame; An, poly(A) tail; 32 K, 32 kDa protein; HE, hemagglutinin/esterase; S, spike protein; 12.7, 12.7 kDa protein; E, envelope protein; M, membrane protein; N, nucleocapsid protein; BCoV, bovine coronavirus; ENO2, enolase II; HRT-18, human rectum tumor-18 cells; DVG, defective viral genome; sgm, subgenomic mRNA; L, leader; c, canonical; nc, noncanonical; TRS, transcription regulatory sequence. ΔL_cTRS_sgm, leader-less sgmRNA derived from canonical TRS; L_ncTRS_sgm, sgmRNA derived from noncanonical TRS; ΔL_ncTRS_sgm, leader-less sgmRNA derived from noncanonical TRS; 5′3′DVG, DVG with sequence elements from 3′ UTR and 5′ UTR; Δ5′DVG, DVG with a sequence element from 3′ UTR; Δ3′DVG, DVG with a sequence element from 5′ UTR; Δ5′3′DVG, DVG lacking sequence elements from 3′ UTR and 5′ UTR

The data collected from the MinION device were base called by Guppy (v5.0.11) with a qscore of 7. The base-called data were first mapped to the host genome (HRT-18 cells: GRCh38; mouse ML cells: GRCm38; mouse liver: GRCm38), yeast enolase II (NC_001140.6) and virus genomes (BCoV: U00735.2; MHV A59: NC_048217.1) using Minimap2 (v2.17-r941) 1 with the parameters “-k 14 -w 1 –splice -u n –MD -a -t 6 –secondary = no” to generate SAM files for analysis of the amount of the coronaviral transcripts in infected cells or mice (Figs. 1B and 5A). The base-called data were also directly mapped to virus genomes (BCoV: U00735.2; MHV: NC_048217.1) using Minimap2 (v2.17-r941) with the parameters “-Y -k 8 -w 1 –splice -g 30,000-G 30000 -F 40000 -N 32 –splice-flank = no –max-chain-skip = 40 -u n –MD -a -t 24 –secondary = no” to generate SAM files for further analysis. Note that the host genome was not removed before mapping to virus genomes. The SAM files, which were generated by mapping to virus genomes using Minimap2 (v2.17-r941) with the parameters described above, were polished by TranscriptClean (v2.0.3) and then transformed into BAM files by SAMtools (v1.15). The resulting BAM files were further processed by bedtool (2.28) to generate BED files followed by R (v4.1.2) for analyses of (i) the amounts of coronavirus transcripts based on the fragment numbers (Figs. 1C and 5B), (ii) the structures and amounts of coronavirus transcripts (Figs. 1D and 5C) and (iii) the reproducibility (Figs. 3C, D, 5D and E). For reproducibility analysis, RNA transcripts with a read count of ≥ 5 were selected, and the reproducibility was measured in reads per kilobase per million mapped sequence reads (RPKM) and determined by Spearman’s correlation coefficient [23].

### Preparation of RNA for biological characterization of noncanonical transcripts

For experimental identification of noncanonical transcripts, HRT-18 cells were infected with BCoV at a multiplicity of infection (MOI) of 0.1, and total cellular RNA was collected at 8, 12, 24 and 48 h postinfection with TRIzol (Thermo Fisher Scientific, Waltham, USA). For reproducibility, the aforementioned procedure was repeated independently with the same BCoV inoculum, and total cellular RNA was collected.

For the experiment with the same HRT-18 cells infected with BCoV from different passages or origins, HRT-18 cells were infected with 0.1 MOI of BCoV inoculum (designated viral passage 0 BCoV; VP0 BCoV). After 48 h of infection, total cellular RNA (VP0 RNA) was harvested, and the supernatant (designated VP1 BCoV) was collected to infect another batch of fresh HRT-18 cells. Total cellular RNA (designated VP1 RNA) was then harvested after 48 h of infection. For the same HRT-18 cells infected with BCoV from different cell origins, HEK-293 T cells were infected with 0.1 MOI of BCoV inoculum (VP0 BCoV). After 48 h of infection, the supernatant (designated VP1 HBCoV) was collected to infect another batch of HRT-18 cells at an MOI of 0.1. Total cellular RNA (designated VP1 HRNA) was then harvested after 48 h of infection. For HRT-18 cells infected with 0.1 and 10 MOI of BCoV inoculum, total cellular RNA (VP0 RNA) was harvested at 16 and 48 h postinfection.

To obtain BCoV-p95 inoculum, HRT-18 cells were infected with BCoV. The infected cells were passaged every third or fourth day depending on the cell condition. The virus collected at 95 days from the supernatant of HRT-18 cells with persistent BCoV infection was designated BCoV-p95 (GenBank: OP296992.1). For the BCoVp95 experiment, the BCoV-p95 isolate (VP0 BCoV-p95) was used as an inoculum to infect fresh HRT-18 cells. Total cellular RNA was then collected (VP0 RNA), and the supernatant (VP1 BCoV-p95) was used to infect another batch of fresh HRT cells. The virus passage step was repeated (VP1-VP6 BCoV-p95) until VP6 RNA was collected. Total cellular RNA collected from VP0, VP4, VP5 and VP6 was used for Northern blotting.

For determination of the synthesis of noncanonical transcripts in mice, 3-week-old male and specific pathogen-free BALB/c mice (BioLASCO Taiwan Co., Ltd.) were infected with 10^6^ PFU of MHV-A59 in 500 µl of DMEM by intraperitoneal inoculation. The livers of MHV-A59-infected mice were collected at 3 days postinfection, and total cellular RNA was prepared.

### Northern blotting assay

For the Northern blotting assay, 10 µg of the collected total cellular RNA was resolved by electrophoresis on a 1% formaldehyde-agarose gel at 150 V for 4 h, and the electrophoresed RNA was transferred to a Nylon Hybond N + membrane (Cytiva Life Sciences, Marlborough, MA, USA) by vacuum blotting for 3 h, followed by UV crosslinking (XL-1000, SpectrolinkerTM), prehybridization and hybridization (NorthernMax™ Kit, Thermo Fisher Scientific, Waltham, USA) at 45 °C for 16 h [24]. The membranes were then probed with 20 pmol of γ-^32^P-5′-end-labeled oligonucleotides (Additional file 1: Table S2), which bind to different positions of the BCoV genome, as indicated in each figure. The probed membranes were exposed to Hyperfilm (Cytiva Amersham) for imaging at -80 °C. The synthesized noncanonical transcripts were quantitated with ImageJ software (NIH, Bethesda, MD).

## Results

### Noncanonical coronavirus transcripts were robustly synthesized and could be categorized into seven populations based on differences in sequence elements

To determine the amounts and structures of the noncanonical bovine coronavirus (BCoV) transcripts in HRT-18 cells, nanopore direct RNA sequencing was employed. Quantification by read counts revealed that ~ 30% of the total cellular RNA was BCoV transcripts (Fig. 1B). The BCoV transcripts consisted of one or more genome fragments (Fig. 1C), and the gene sequences of the fragments were identical to those from different portion(s) of the full-length genome. In addition, the BCoV transcripts could be divided into two main categories, canonical transcripts (Fig. 1E) and noncanonical transcripts (Fig. 1F), with various quantities (Fig. 1D), based on the difference in sequence elements and the potential synthesis mechanisms. The canonical transcripts were well-established canonical coronavirus sgmRNAs (Fig. 1E) with a leader sequence derived from well-defined canonical TRS-Bs (cTRS, Fig. 1A) located immediately upstream of each structural and accessory protein-encoding gene and are thus defined as canonical sgmRNAs (Fig. 1E). Accordingly, TRS-Bs, which shared sequence homology with canonical TRS-Bs but were not located immediately upstream of each structural and accessory protein-encoding gene, are defined as noncanonical TRS-Bs (ncTRS, Fig. 1A). In addition, it has been suggested that during coronavirus replication, a defective viral genome (DVG), which is a truncated version of the genome, can be synthesized irrespective of TRSs [12]. Consequently, based on whether the structures of noncanonical transcripts are relevant to TRSs, noncanonical transcripts were categorized into 2 subcategories: noncanonical sgmRNAs and DVGs (Fig. 1F). The method used for this classification is explained in Additional file 1: Figure S1 and the associated figure legend. In the noncanonical sgmRNA subcategory (Fig. 1F, upper panel), based on whether the coronavirus sgmRNAs had or did not have a leader and whether they were derived from canonical or noncanonical TRS-Bs, the sgmRNAs were further divided into three populations, including sgmRNAs without a leader but with a 5′ sequence identical to the flanking sequence of a canonical TRS-B (1. ΔL_cTRS_sgm), sgmRNAs with a leader but derived from a noncanonical TRS-B (2. L_ncTRS_sgm) and sgmRNAs without a leader but with a 5′ sequence identical to the flanking sequence of a noncanonical TRS-B (3. ΔL_ncTRS_sgm). In the DVG subcategory [25] (Fig. 1F, lower panel), DVGs were further divided into four populations based on the presence of sequence elements from 3′ UTR or/and 5′ UTR, including DVGs with sequence elements from 3′ UTR and 5′ UTR (1. 5′3′DVG), DVGs with a sequence element from 5′ UTR (2. Δ3′DVG), DVGs with a sequence element from 3′ UTR (3. Δ5′DVG) and DVGs lacking sequence elements from 3′ UTR and 5′ UTR (4. Δ5′3′DVG). Consequently, based on the classification, the RNA transcripts consisting of 1 fragment from one part of the genome shown in Fig. 1C included 5′3′DVG Δ5′DVG, Δ3′DVG, Δ5′3′DVG, ΔL_cTRS_sgm and ΔL_ncTRS_sgm; the RNA transcripts consisting of 2 fragments from two different parts of the genome included 5′3′DVG, Δ5′DVG, Δ3′DVG, Δ5′3′DVG, canonical sgmRNA and L_ncTRS_sgm; the RNA transcripts consisting of more than 2 fragments (3, 4, 5 and ≥ 6 in Fig. 1C) from different parts of the genome included 5′3′DVG, Δ5′DVG, Δ3′DVG and Δ5′3′DVG.

Based on the results derived from nanopore direct RNA sequencing, it is concluded that (i) in addition to the previously well-defined genomes (Fig. 1A) and canonical sgmRNAs (Fig. 1E), noncanonical transcripts are also synthesized (Fig. 1F) at high abundance (Fig. 1D), and (ii) noncanonical transcripts can be further divided into 2 subcategories: noncanonical sgmRNAs (3 populations, Fig. 1F, upper panel) and DVGs (4 populations, Fig. 1F, lower panel).

### Coronaviruses may employ a variety of mechanisms to synthesize viral RNAs

**Fig. 2:**
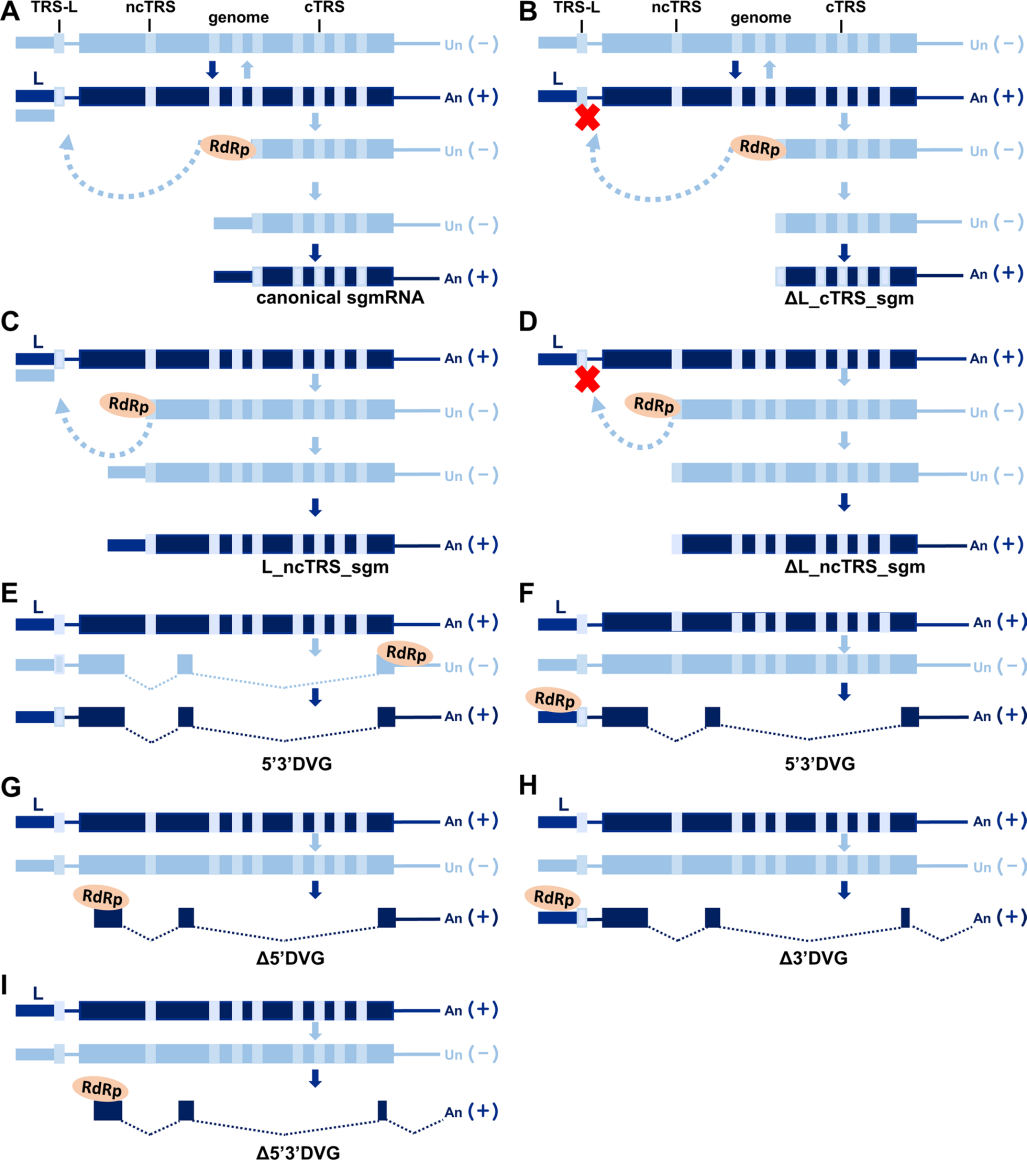
Coronaviruses employ a variety of mechanisms to synthesize their viral RNAs. **A**–**I** Schematic diagram depicting the mechanism of RNA synthesis for each group of coronavirus transcripts shown in Fig. 1. For detailed explanations regarding the mechanisms, please see the text. ncTRS, noncanonical TRS-B; cTRS, canonical TRS-B. L, leader; c, canonical; nc, noncanonical; TRS, transcription regulatory sequence; TRS-L, leader TRS, ncTRS, noncanonical TRS; cTRS, canonical TRS. RdRp, RNA-dependent RNA polymerase; sgm, subgenomic mRNA; DVG, defective viral genome; ΔL_cTRS_sgm, leader-less sgmRNA derived from canonical TRS; L_ncTRS_sgm, sgmRNA derived from noncanonical TRS; ΔL_ncTRS_sgm, leader-less sgmRNA derived from noncanonical TRS; 5′3′DVG, DVG with sequence elements from 3′ UTR and 5′ UTR; Δ5′DVG, DVG with a sequence element from 3′ UTR; Δ3′DVG, DVG with a sequence element from 5′ UTR; Δ5′3′DVG, DVG lacking sequence elements from 3′ UTR and 5′ UTR

While analyzing the structures of noncanonical BCoV transcripts (Fig. 1), it is speculated that coronaviruses may apply various known strategies employed by RNA viruses [5, 26–29] to synthesize their viral RNA. These mechanisms are shown in Fig. 2 based on the coronaviral RNA structures shown in Fig. 1. Note that only the mechanism for coronavirus canonical sgmRNA synthesis (Fig. 2A) has been well established [30–32], and the synthesis mechanisms for coronavirus noncanonical transcripts have not previously been studied. Thus, the delineated synthesis mechanisms for noncanonical transcripts proposed below (Fig. 2B–I) are based on the data shown in Fig. 1. For synthesis of canonical sgmRNAs (Fig. 2A), coronavirus RNA-dependent RNA polymerase (RdRp) initiates (−)-strand sgmRNA synthesis, attenuates at the canonical TRS-B, switches the template to the TRS-L and acquires the leader to synthesize the (−)-strand sgmRNA from which the (+)-strand canonical sgmRNA with the leader is made. For the (+)-strand canonical sgmRNA without the leader (ΔL_cTRS_sgm, Fig. 2B), it is speculated that, if coronavirus RdRp does not switch the template and dissociate near or at the canonical TRS-B, the synthesis of the (−)-strand leaderless-canonical sgmRNA from which the (+)-strand canonical sgmRNA without the leader is made occurs (ΔL_cTRS_sgm, Fig. 2B). Based on the RNA structure, it has also been proposed that the synthesis of the noncanonical sgmRNA with a leader (L_ncTRS_sgm, Fig. 2C) is performed via a similar strategy to that of the canonical sgmRNA (Fig. 2A) but attenuates the noncanonical TRS-B. Similarly, if coronavirus RdRp does not switch the template and dissociate near or at noncanonical TRS-B, the synthesis of the (−)-strand leader-less noncanonical sgmRNA from which the (+)-strand noncanonical sgmRNA without the leader is made occurs (ΔL_ncTRS_sgm, Fig. 2D).

How coronavirus DVGs are synthesized remains unknown; however, based on the difference in sequence elements shown in Fig. 1, the synthesis mechanisms for different DVGs are proposed as follows. For 5′3′DVG (Figs. 2E and F), coronavirus RdRp may initiate (−)-or (+)-strand synthesis at one end of the genome using the (+)-or (−)-strand as a template, respectively, switch the template(s) and acquire the sequence at the other end, synthesizing 5′3′DVG with sequence elements from 3′ UTR and 5′ UTR. Based on the current knowledge that the sequence elements from 3′ UTR and 5′ UTR harbor replication-required *cis*-acting elements, it is assumed that DVGs without sequence elements from 3′ UTR and 5′ UTR cannot replicate; thus, (+)-strand DVGs are the end products. Consequently, it is speculated that when coronavirus RdRp internally initiates (+)-strand synthesis using the (−)-strand genome as a template and acquires the sequence at the 3′ UTR to finish (+)-strand synthesis with or without template switching, (+)-strand Δ5′DVG with a 3′ but lacking a 5′ UTR is produced (Fig. 2G). In line with this mechanism, the sequence or secondary structure to which RdRp binds may serve as an initiator. On the other hand, coronavirus RdRp uses the (−)-strand genome as a template, initiates (+)-strand synthesis at the 3′ end of the (−)-strand genome, synthesizes the (+)-strand with or without template switching and dissociates from the (−)-strand template to acquire a poly(A) tail, synthesizing (+)-strand poly(A)-containing Δ3′DVG with a 5′ but lacking a 3′ UTR (Fig. 2H). Finally, when coronavirus RdRp internally initiates (+)-strand synthesis using the (−)-strand genome as a template and then dissociates from the (−)-strand template to acquire a poly(A) tail, (+)-strand Δ5′3′DVG with a poly(A) tail but without sequence elements from 3′ UTR and 5′ UTR is synthesized (Fig. 2I).

### Noncanonical coronavirus transcripts are experimentally validated and are largely reproducible

**Fig. 3:**
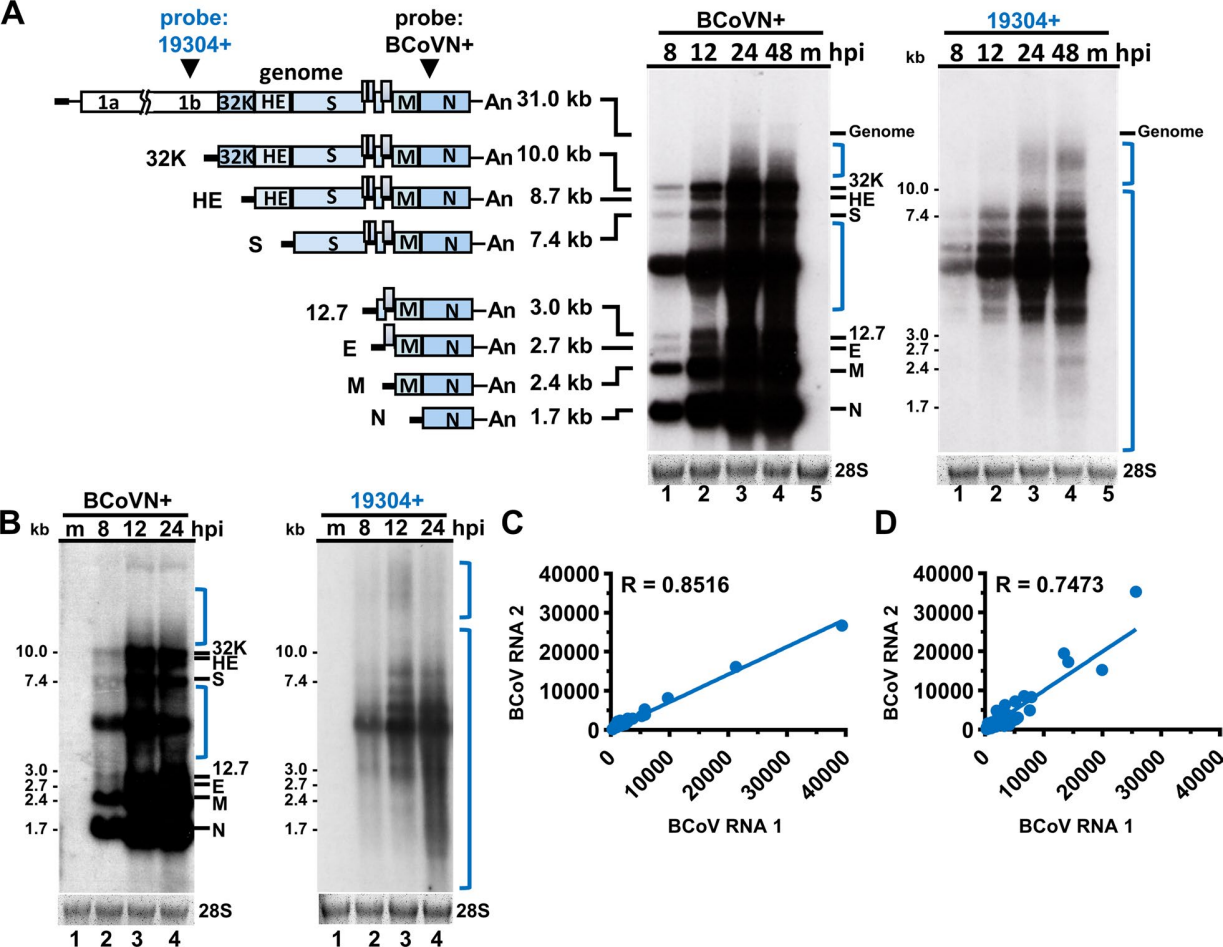
Noncanonical coronavirus transcripts are experimentally validated and reproducible. **A** Left panel: Diagram depicting the BCoV genome and canonical sgmRNAs. Middle and right panels: Detection of noncanonical transcripts by Northern blotting. HRT-18 cells were infected with BCoV at an MOI of 0.1, and total cellular RNA was collected at 8, 12, 24 and 48 h postinfection (hpi). In addition to the genome and canonical sgmRNAs (denoted by black bars, middle panel), signals representing noncanonical transcripts (denoted by blue brackets, middle and right panels) were detected by Northern blotting with the ^32^P-labeled primer probes BCoVN + (middle panel) and 19,304 + (right panel). **B** Reproducibility of noncanonical transcripts (denoted by blue brackets) detected by Northern blotting. **C**–**D** Reproducibility of canonical transcripts (**C**) and noncanonical transcripts (**D**) from two biological replicates. The dots in (**C**) and (**D**) correspond to the read numbers of certain noncanonical transcript species measured by nanopore direct RNA sequencing with RNA samples (RNA 1 and RNA2) collected from two independent experiments of virus infection. The noncanonical transcript species with a read count of ≥ 5 were selected from RNA samples: RNA1 and RNA2, and the dot was mapped according to the reads measured from RNA1 (X-axis) and RNA 2 (Y-axis). The reproducibility was measured in reads per kilobase per million mapped sequence reads (RPKM) and evaluated by Spearman’s correlation coefficient. R = 0.000–0.3999, low reproducibility; R = 0.4000–0.5999, moderate reproducibility; R = 0.6000–1.0000, high reproducibility. kb, kilobase; m, mock-infected cells; hpi, hours postinfection. 28S, 28S rRNA; An, poly(A) tail; 32 K, 32 kDa protein; HE, hemagglutinin/esterase; S, spike protein; 12.7, 12.7 kDa protein; E, envelope protein; M, membrane protein; N, nucleocapsid protein

Based on the in silico analysis by nanopore direct RNA sequencing (Fig. 1), it was suggested that noncanonical coronavirus transcripts can be synthesized. To experimentally validate the results, in addition to genome and canonical sgmRNAs, other coronaviral transcripts (that is, noncanonical transcripts) were also synthesized during infection, and as a complementary method, the Northern blotting assay, which can be used to directly detect and quantitate RNA without further processing, was employed. With the ^32^P-labeled primer probe BCoVN + (Fig. 3A, left panel), in addition to signals representing the genomes and canonical sgmRNAs, multiple signals with different sizes and intensities were also observed (Fig. 3A, middle panel, denoted by blue brackets). Furthermore, the amounts of multiple signals increased with the time of infection. Therefore, along with the results obtained from nanopore direct RNA sequencing, these signals represented previously unidentified coronaviral RNA species. Multiple signals with different sizes and intensities were also observed at different hours postinfection (hpi) (Fig. 3A, right panel, denoted by blue brackets) with ^32^P-labeled primer probe 19,304 + (Fig. 3A, left panel), which were previously predicted to probe only the genome, further suggesting that these signals represent previously unidentified coronaviral RNA species during coronavirus infection. Based on the results above and those obtained from nanopore direct RNA sequencing (Fig. 1), it is concluded that these previously unidentified coronaviral RNA species determined by Northern blotting represent noncanonical coronavirus transcripts, experimentally validating the synthesis of noncanonical coronavirus transcripts. Note that due to the diverse genome structures of noncanonical transcripts, it is possible that there are multiple RNA species synthesized at a certain time point postinfection, but only RNA species with sequences corresponding to primer probes, such as BCoVN(+) and 19,304(+), could be detected by Northern blotting (Fig. 3A, middle and right panel).

Whether the noncanonical transcripts in coronaviruses are opportunistically synthesized or reproducible remains unknown. To this end, the same BCoV inoculum used for the experiment shown in Fig. 3A was employed to infect another batch of HRT-18 cells, and the collected RNA was detected with the same ^32^P-labeled primer probes. As shown in Fig. 3B, left and right panels, similar patterns of Northern blotting signals were identified in comparison with those shown in Fig. 3A, experimentally suggesting that the synthesis of noncanonical transcripts is reproducible. This conclusion was supported by the analysis based on nanopore direct.

RNA sequencing data in which both canonical (R = 0.8516) and noncanonical transcripts (R = 0.7473) were largely reproducible (Fig. 3C and D). Since it is suggested that canonical sgmRNAs and DVGs with 5′- and 3′-terminal sequences can be packaged into virions [13, 33], the detected viral RNA species can be derived either from packaged DVG and sgmRNA species or from the full-length genome, as depicted in Fig. 2. However, based on the results shown in Fig. 3B and D, under regular coronavirus infection with the same BCoV inoculum and cells, the synthesis of noncanonical transcripts is largely reproducible (R = 0.7473) regardless of which viral RNA species in virions are used as templates for the synthesis of noncanonical transcripts in infected cells. Thus, under regular coronavirus infection with the same BCoV inoculum and cells, the synthesis of noncanonical transcripts is reproducible overall, further suggesting the biological significance of noncanonical transcripts during coronavirus infection.

### Species and amounts of noncanonical transcripts are altered under different infection conditions

**Fig. 4:**
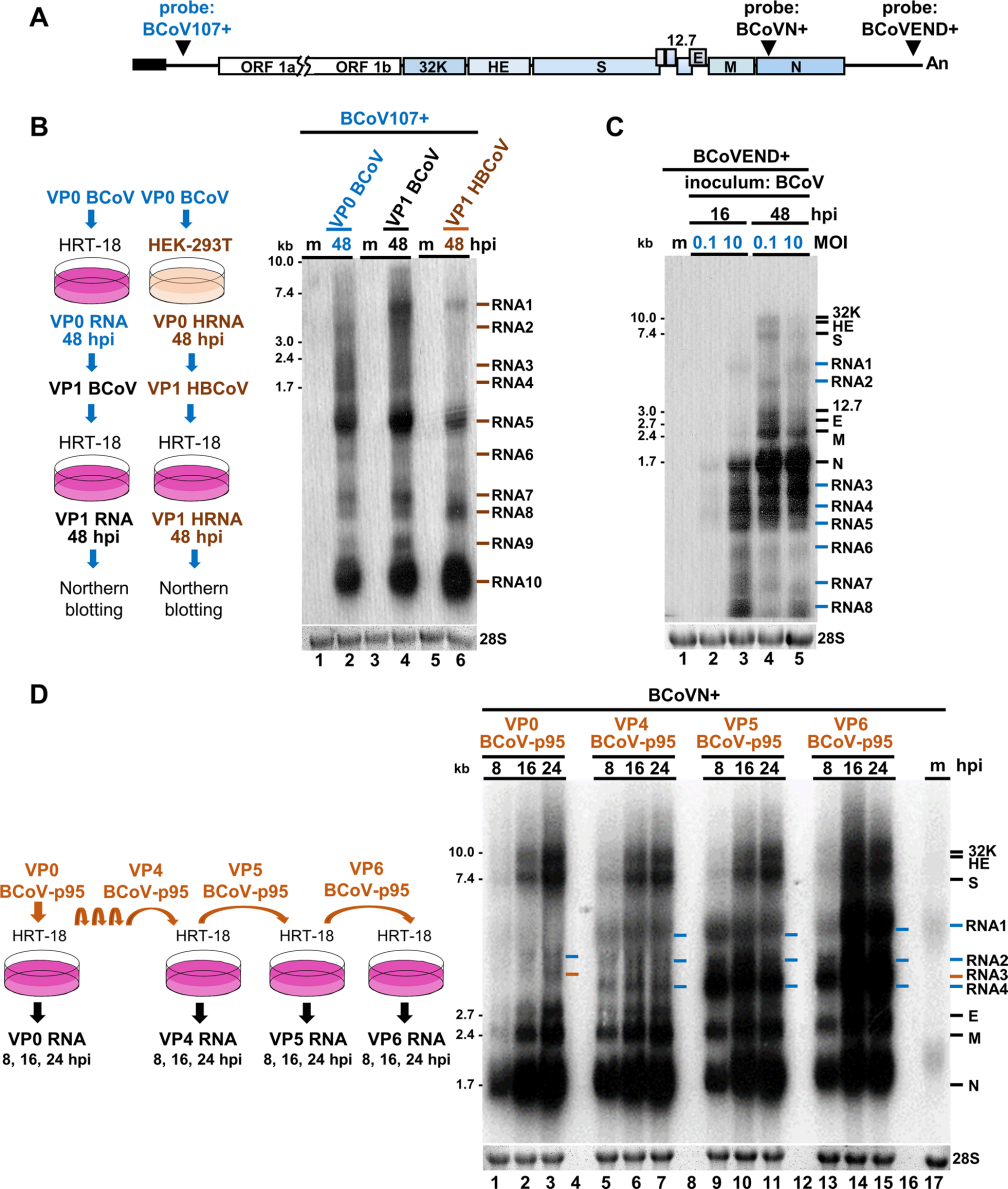
Synthesis of noncanonical coronavirus transcripts is regulated under different infection conditions. **A** Diagram depicting the probes used for Northern blotting in Figures (**B**–**D**). **B** Left panel: Diagram depicting the preparation of BCoV inoculum from different passages and origins. For the BCoV inoculum from different passages, HRT-18 cells were infected with 0.1 MOI of BCoV inoculum (designated viral passage 0 BCoV; VP0 BCoV). After 48 h of infection, total cellular RNA (VP0 RNA) was harvested, and the supernatant (designated VP1 BCoV) was collected to infect another batch of fresh HRT-18 cells. For BCoV inoculum from different cell origins, HEK-293T cells were infected with 0.1 MOI of BCoV inoculum (VP0 BCoV). After 48 h of infection, the supernatant (designated VP1 HBCoV) was collected to infect another batch of HRT-18 cells at an MOI of 0.1. Right panel: Detection of noncanonical transcripts from HRT-18 cells infected with BCoV from different passages or origins by Northern blotting. The RNA species representing noncanonical transcripts between passages (Lanes 2 and 4) and origins (Lanes 4 and 6) are denoted by brown bars. **C** Detection of noncanonical transcripts from the same HRT-18 cells infected with different MOIs (0.1 and 10) of BCoV by Northern blotting. The RNA species representing noncanonical transcripts between different MOIs (Lanes 4 and 5) at 48 hpi are denoted by blue bars. **D** Comparison of the species and amounts of noncanonical transcripts between passages from fresh HRT-18 cells infected with the BCoV-p95 isolate. Left panel: Diagram depicting different passages of RNA collected from fresh HRT-18 cells infected with BCoV-p95 inoculum (VP0 BCoV-p95) and supernatant from different passages of BCoV-p95 (VP1-VP6 BCoV-p95). Right panel: Detection of noncanonical transcripts from the aforementioned total cellular RNA by Northern blotting. The RNA species representing noncanonical transcripts are denoted by orange and blue bars. kb, kilobase; m, mock-infected cells; hpi, hours postinfection; 28S, 28S rRNA

Whether noncanonical transcripts are still reproducible under different infection conditions has not yet been documented. Because (i) Northern blotting can detect and quantitate noncanonical transcripts and (ii) the aim of the experiment was simply to examine whether the synthesized RNA patterns that represent the noncanonical transcripts (but not the specific RNA population) were altered under different infection conditions, a Northern blotting assay was selected for this aim. Consequently, it is hypothesized that, based on the RNA patterns detected by Northern blotting assay, whether the species and amounts are altered under different infection conditions can then be determined. We first tested whether noncanonical transcripts were reproducible when the same HRT-18 cells were infected with BCoV from different passages or origins. For this, HRT-18 cells were infected with BCoV inoculum, which was designated viral passage 0 BCoV (VP0 BCoV) (Fig. 4B, left panel). After 48 h of infection, RNA (VP0 RNA) was harvested, and the supernatant (designated VP1 BCoV) was collected to infect another batch of HRT-18 cells, which were then harvested after 48 h of infection (designated VP1 RNA). As shown in Fig. 4B, right panel, Lanes 2 and 4, the patterns representing noncanonical transcript species between passages VP0 and VP1 were highly similar but displayed different intensities (Additional file 1: Figure S2A, denoted by blue bars (VP0) and black bars (VP1)) by Northern blotting with the probe BCoV107 + . Similar results were also observed between inoculums obtained from supernatant collected from BCoV VP0-infected HRT-18 (VP1 BCoV) and HEK-293T cells (VP1 HBCoV) (Fig. 4B, right panel, Lanes 4 and 6, respectively, and Additional file 1: Figure S2A, denoted by brown bars). The results suggest that noncanonical transcripts are largely reproducible, but in different amounts, when the same HRT-18 cells are infected with BCoV from different passages or origins. Similar results were also obtained when different MOIs of inoculum were used to infect HRT-18 cells (Fig. 4C, Lanes 4 and 5, and Additional file 1: Figure S2B, denoted by blue bars) with the probe BCoVEND + . Together, these results suggest that the synthesis of noncanonical transcripts is reproducible, but the amount is regulated under different infection conditions.

We next examined whether both the species and amounts of noncanonical transcripts varied between different virus passages from fresh HRT-18 cells infected with BCoV-p95 inoculum (VP0 BCoV-p95) and with supernatant from different passages of BCoV-p95 (VP1-VP6 BCoV-p95) (Fig. 4D, left panel). The BCoV-p95 isolate (GenBank: OP296992.1) is a BCoV variant with an altered genome structure of 106 nucleotide mutations obtained from the supernatant of HRT-18 cells persistently infected with BCoV and was used as an inoculum (VP0 BCoV-p95) in this experiment. As shown in Fig. 4D, right panel, it was found that (i) the species of noncanonical transcripts differed between VP0 and VP 4 and (ii) the species of noncanonical transcripts were similar among VP4, VP5 and VP6 (Fig. 4D, right panel), but the amounts were different (Additional file 1: Figure S2C). By comparison of the genome structure between BCoV and the variant BCoV-p95, it was found that 47 nucleotides in BCoV were mutated from AU to GC and 41 nucleotides from GC to AU. Some of these mutations also occurred around the recombination points during the synthesis of noncanonical transcripts. Consequently, based on a previous study showing that AU-rich sequences can affect recombination efficiency and thus the synthesized RNA species [34], mutation may be one of the reasons for the different patterns of RNA species found between BCoV and BCoV-p95. Consequently, the results suggest that both the species and amounts of noncanonical transcripts varied among different virus passages of BCoV-p95-infected HRT-18 cells.

Together, the results suggest that the species or/and amounts of noncanonical transcripts can be regulated under different infection conditions, including (i) coronavirus inoculum isolated from different cells (BCoV from HRT-18 cells vs. HBCoV from HEK-293T cells, Fig. 4B), from different environments (BCoV from HRT-18 cells vs. BCoV-p95 from HRT-18 cells with persistence, Fig. 4D) and from different passages (VP0-VP1, Fig. 4B; VP0-VP6, Fig. 4D), (ii) coronavirus inoculum with altered genome structure (BCoV-p95, Fig. 4D) and (iii) coronavirus inoculum with different MOIs (0.1 vs. 10, Fig. 4C).

### The biological features of noncanonical coronavirus transcripts in vitro and in vivo are similar

**Fig. 5:**
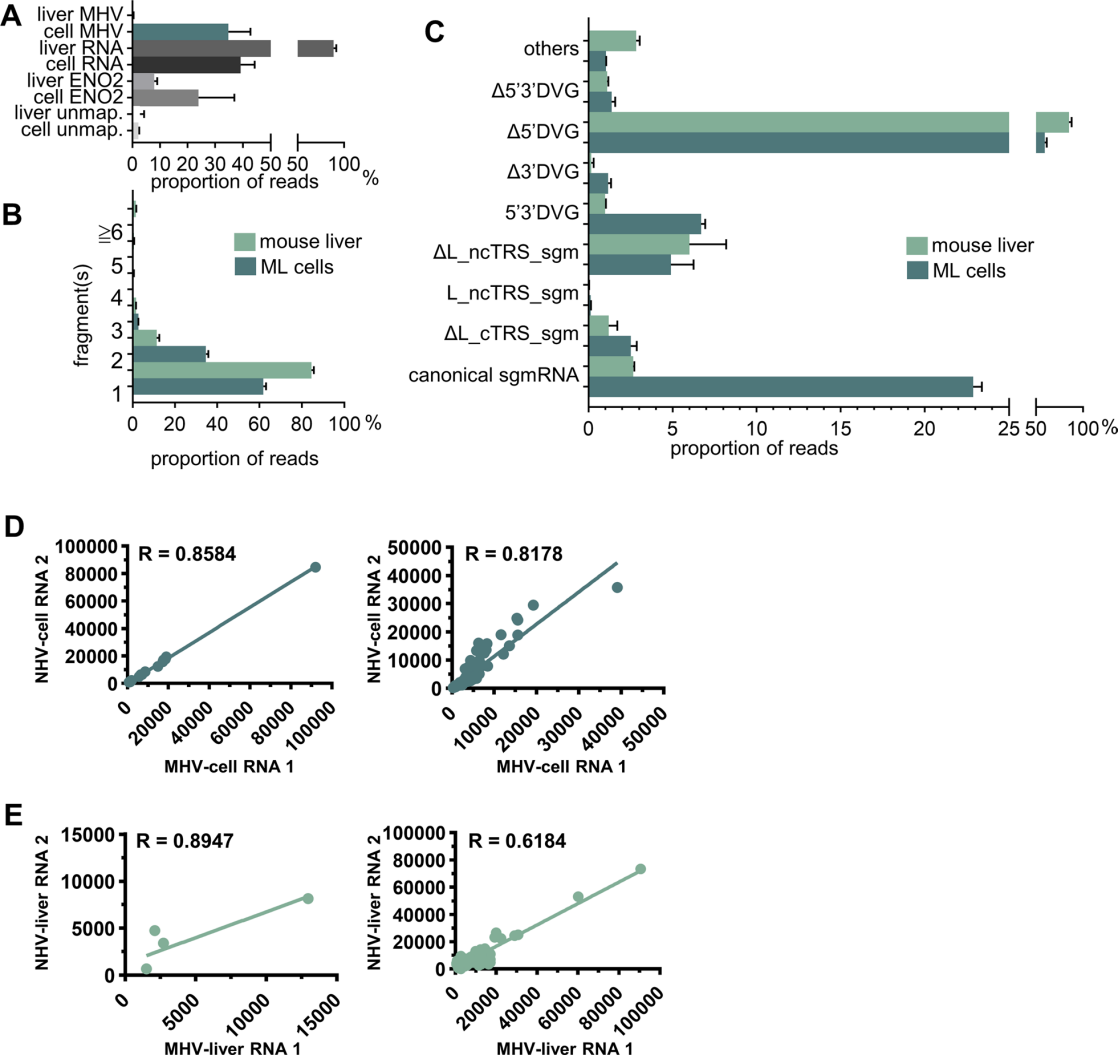
Comparison of the biological nature of noncanonical coronavirus transcripts between in vitro and in vivo conditions. **A** Comparison of the amounts of MHV-A59 transcripts between virus-infected ML cells and mice. cell, ML cells; liver, mouse liver; ENO2, yeast enolase II mRNA; unmap, unmapped. **B** Comparison of the ratio among transcripts based on the fragment numbers of which the transcripts were composed between ML cells and mice. **C** Comparison of the ratio of individual MHV-A59 transcripts among the whole transcripts between ML cells and mice. **D**–**E** Reproducibility of canonical (left panel) and noncanonical (right panel) transcripts with a read count of ≥ 5 in MHV-A59-infected ML cells (**D**) and mice (**E**). The reproducibility was measured as RPKM values and evaluated by Spearman’s correlation coefficient. The data in (**A**–**C**) represent the means of two independent experiments. MHV, mouse hepatitis virus-A59; ENO2, Enolase II; unmap, unmapped; DVG, defective viral genome; sgm, subgenomic mRNA; L, leader; c, canonical; nc, noncanonical; ML cells, mouse L cells. ΔL. cTRS. sgm, leader-less sgmRNA derived from canonical TRS; ΔL_cTRS_sgm, leader-less sgmRNA derived from canonical TRS; L_ncTRS_sgm, sgmRNA derived from noncanonical TRS; ΔL_ncTRS_sgm, leader-less sgmRNA derived from noncanonical TRS; 5′3′DVG, DVG with sequence elements from 3′ UTR and 5′ UTR; Δ5′DVG, DVG with a sequence element from 3′ UTR; Δ3′DVG, DVG with a sequence element from 5′ UTR; Δ5′3′DVG, DVG lacking sequence elements from 3′ UTR and 5′ UTR

To examine whether the characteristics of noncanonical transcripts in vivo are similar to those in cells, the same amounts of sample acquired from MHV-A59-infected ML cells and MHV-59-infected mouse liver were used for nanopore RNA direct sequencing. As shown in Fig. 5A, the relative amounts of MHV-59 transcripts synthesized in the liver were low (~ 0.4% of total cellular RNA) in comparison with those in ML cells (~ 35% of total cellular RNA). In addition, MHV-59 transcripts from both ML cells and the liver consisted of 1 or more fragments, and the gene sequences of the fragments were identical to those from different portions of the full-length genome (Fig. 5B). Although the amounts of MHV-A59 transcripts in the liver were relatively low, further analyses suggested that noncanonical transcripts can also be synthesized in the liver of MHV-59-infected mice, as evidenced by the number of reads for each transcript from mouse liver and ML cells infected with MHV-A59 shown in Additional file 1: Table S1. Consequently, the noncanonical transcripts can also be categorized into 2 subcategories: noncanonical sgmRNAs and DVGs; however, the ratio of each classified noncanonical transcript between MHV-59-infected ML cells and mouse liver varied (Fig. 5C) based on the data shown in Additional file 1: Table S1.

The lower amounts of transcripts synthesized in vivo were expected because after infection, MHV-59 was distributed in different tissues and organs, and thus, the number of viral RNAs per cell in mice was much lower than that in ML cells. Furthermore, nanopore direct RNA sequencing showed that both canonical transcripts (Fig. 5D and E, left panel) and noncanonical transcripts (Fig. 5D and E, right panel) derived from MHV-59-infected ML cells (Fig. 5D) and mouse liver (Fig. 5E) were reproducible overall. In conclusion, noncanonical coronavirus transcripts can also be synthesized in vivo. In addition, the biological features of noncanonical coronavirus transcripts were similar between in vitro and in vivo conditions.

## Discussion

Due to the diverse structures and lengths of noncanonical coronavirus transcripts, their synthesis has not been experimentally validated, nor has their biology been revealed both in vitro and in vivo. In the current study, the synthesis of coronavirus noncanonical transcripts was suggested by nanopore direct RNA sequencing and experimentally validated by Northern blotting. Their biological features were also experimentally characterized. The limitations and biological significance of the study are discussed below.

Nanopore direct RNA sequencing is an excellent tool that allows us to comprehensively determine the abundance and diverse structures of coronavirus RNA species, especially those with low copy numbers and without previously identified sequences as references for analysis (Fig. 1). However, because SuperScript™ III reverse transcriptase (cat No. 18080044, Thermo Fisher Scientific, Waltham, USA), which is optimized to synthesize first-strand cDNA of up to ~ 12 kb, was used for nanopore direct RNA sequencing, the results may not cover all coronavirus transcripts, especially those with longer sizes. On the other hand, Northern blotting, which can detect viral RNA without amplification, could be used to experimentally detect the coronavirus transcripts of different lengths, including the ~ 30 kb full-length genome. In addition, RNA detected by Northern blotting can be visualized and quantitated. However, the Northern blotting results did not specify the corresponding population of the noncanonical transcripts. Thus, to comprehensively determine the abundance and structures of specific coronavirus RNA species, especially those with low copy numbers and without previously identified sequences as references for analysis, nanopore direct RNA sequencing was employed (Fig. 1). On the other hand, if the experiments are conducted simply to examine whether, in addition to the genome and canonical sgmRNAs, other coronaviral RNA species (that is, noncanonical transcripts) can also be synthesized (Fig. 3) and to simply examine whether the overall amounts and species of noncanonical transcripts are altered under different infection conditions based on the RNA patterns (Fig. 4), in addition to nanopore direct RNA sequencing, Northern blotting can also be employed. Consequently, it is suggested that with nanopore direct RNA sequencing and biochemistry tools such as Northern blotting, the landscape and biological features of coronavirus noncanonical transcripts can be more comprehensively determined.

It has also been suggested that the synthesis of sgmRNAs without a leader is correlated with the TRS and the structure near the TRS [28, 29]. Consequently, it is speculated that the difference in the number of TRSs (and their variants) and structures between the BCoV and MHV genomes, which can lead to the synthesis of sgmRNAs with or without leader sequences, may be the factor determining the synthesis of different portions of sgmRNAs between BCoV (Fig. 1D) and MHV (Fig. 5C and Additional file 1: Table S1). Furthermore, due to the large size of the coronavirus genome (~ 30 kb) and technical limitations such as reverse transcriptase, it is possible that reverse transcriptase reads long viral RNA transcripts for cDNA synthesis and dissociates halfway, leading to the synthesis of many incomplete reads with missing sequences at the termini. This leads to the question of how the missing leader and terminal sequences of transcripts can be identified from the nanopore sequencing reads. As described in the results, based on whether the 5′ terminal sequence is relevant to the TRS, the transcripts are classified into TRS-relevant transcripts and TRS-irrelevant transcripts (that is, DVGs). In terms of the transcripts (ΔL_cTRS_sgm, ΔL_ncTRS_sgm, Δ5′DVG and Δ5′3′DVG) lacking 5′ terminal sequences, including the leader sequence, if the transcripts had no 5′UTR sequence but contained the sequences positioned within the 50 nucleotides of cTRS-B or ncTRS-B at their 5′ termini, they were classified as ΔL_cTRS_sgm and ΔL_ncTRS_sgm, respectively; in contrast, if the transcripts lacked the 5′ UTR sequences and their 5′ terminal sequences were not relevant to the TRS, the transcripts were classified as Δ5′DVG or Δ5′3′DVG. On the other hand, according to the synthesis mechanism of sgmRNA, it was defined during the classification (Additional file 1: Figure S1) that the synthesis of sgmRNA must contain 3′ UTR. Consequently, in terms of the transcripts (Δ3′DVG and Δ5′3′DVG) lacking a sequence from 3′ UTR, if the transcripts contained partial or complete 5′ UTR sequences but lacked the 3′ UTR sequence, the transcripts were classified as Δ3′DVG; if the transcripts lacked both 5′ and 3′ UTR sequences, the transcripts were classified as Δ5′3′DVG. Consistent with this, the synthesis capability of reverse transcriptase can also explain why an elevated proportion of Δ5'DVG was observed in BCoV-infected HRT-18 cells (Fig. 1D), MHV-59-infected ML cells and mouse liver (Fig. 5C).

Based on the results shown in Fig. 3, under regular coronavirus infection with the same BCoV inoculum and cells, the synthesis of noncanonical transcripts is reproducible overall. Thus, the biological characteristics of abundance and reproducibility (Figs. 1 and 3) further highlight the biological significance of noncanonical transcripts during coronavirus infection. On the other hand, it was also found that the species and amounts of noncanonical transcripts were regulated under different infection conditions (Fig. 4). Therefore, it is possible that coronaviruses may have the potential to regulate the species and amounts of noncanonical transcripts in response to altered infection conditions for viral fitness, and such regulatory roles may also play important roles in pathogenesis. In addition, because noncanonical coronavirus transcripts can also be synthesized in vivo, identification of the function of noncanonical transcripts derived in vivo is also a priority in further understanding their roles in coronavirus pathogenesis in vivo. Consequently, the current results may provide further opportunities for studying the biological functions of noncanonical transcripts and the mechanisms by which coronaviruses regulate the synthesis of noncanonical transcripts. The outcomes of these studies may contribute to the development of antiviral strategies.

Of the coronaviral RNA synthesis mechanisms illustrated in Fig. 2, most require a copy-choice template-switching recombination step. In addition, recombination between coronavirus RNA species during natural infections has been documented [35, 36]. Since there are substantial amounts of noncanonical coronavirus transcripts synthesized during infection, recombination between noncanonical transcripts and the error-prone largest known RNA genome may assist coronaviruses in overcoming error catastrophe and thus restoring the fitness of the genome under selection pressure. Furthermore, it has also been demonstrated that the longer coronaviral RNA transcript with TRSs can serve as a template for shorter sgmRNA synthesis [7]. Therefore, the longer canonical or noncanonical sgmRNAs with TRSs identified in the current study may be templates for shorter sgmRNA or/and DVG synthesis. Similarly, longer DVGs with TRSs could also be templates for the synthesis of shorter sgmRNAs and/or shorter DVGs. Accordingly, such a strategy may relieve the pressure on the genome and increase the species variety of noncanonical transcripts.

In this study, we (i) experimentally determined the synthesis of noncanonical transcripts, (ii) demonstrated that the species and amounts of noncanonical transcripts are largely reproducible during regular infection but can be regulated under altered infection environments and (iii) found that the characteristics of noncanonical transcripts in vivo are similar to those in cell culture. The biological significance based on the findings is as follows: (i) classification of experimentally validated noncanonical transcripts extends the current model for coronavirus gene expression; (ii) synthesis of a variety of noncanonical transcripts may assist the coronavirus genome in overcoming error catastrophe via its recombination with the transcripts under harsh environments; and (iii) the regulated noncanonical transcripts in terms of species and amounts under different environments may have the potential to contribute to the pathogenesis of coronaviruses. Consequently, the identified biological characteristics of noncanonical coronavirus transcripts may lead to further research questions, including (i) what are the molecular mechanisms driving the synthesis of noncanonical transcripts? (ii) How are noncanonical transcripts regulated in response to altered infection conditions? (iii) How do alterations of populations in noncanonical transcripts impact virus evolution and adaptation to new hosts? (iv) How do host factors impact the synthesis and activity of noncanonical transcripts? Although the current study on coronavirus noncanonical transcripts may pose challenges in the control of coronavirus diseases, the answers obtained from the aforementioned studies may contribute to the development of antiviral strategies.

## Conclusions

In the current study with BCoV and MHV-A59, we experimentally validated the synthesis and biological characteristics of noncanonical coronavirus transcripts both in vitro and in vivo. The identified features of noncanonical transcripts in terms of abundance, reproducibility and variety extend the current model for coronavirus gene expression. The capability of coronaviruses to regulate the species and amounts of noncanonical transcripts may contribute to the pathogenesis of coronavirus during infection. The identification of the biological characteristics of noncanonical coronavirus transcripts may assist the coronavirus research community in answering previously unanswered questions on coronavirus gene expression and pathogenesis and provide a database for a variety of biomedical studies.

### Supplementary Information

**Additional file 1** of Biological characterization of coronavirus noncanonical transcripts in vitro and in vivo.

## Data Availability

The sequencing data are deposited into the Open Science Framework (OSF) at https://osf.io/cm7z6/. Code for the analyses described in this study is available at https://github.com/BJ-Chen-Eric/The-biology-of-coronavirus-noncanonical-transcripts-in-vitro-and-in-2-vivo/tree/main.

## Abbreviations

CoV: Coronavirus
SARS-CoV-2: Severe acute respiratory syndrome coronavirus 2
UTR: Untranslated region
ORF: Open reading frame
sgmRNA: Subgenomic mRNA
TRS: Transcription regulatory sequence
DVG: Defective viral genome
MHV: Mouse hepatitis viruses
BCoV: Bovine coronavirus
HRT-18 cells: Human rectal tumor-18 cells
HEK-293T cells: Human embryonic kidney-293T cells
ML cells: Mouse L cells
MOI: Multiplicity of infection
ENO2: Yeast enolase II mRNA
YHR174W: Yeast enolase II
RPKM: Reads per kilobase per million mapped sequence reads
VP: Virus passage
cTRS: Canonical TRS-B
ncTRS: Noncanonical TRS-B
RdRp: RNA-dependent RNA polymerase
ΔL_cTRS_sgm: Leader-less sgmRNA derived from canonical TRS
L_ncTRS_sgm: SgmRNA derived from noncanonical TRS
ΔL_ncTRS_sgm: Leader-less sgmRNA derived from noncanonical TRS
5′3′DVG: DVG with sequence elements from 3′ UTR and 5′ UTR
Δ5′DVG: DVG with a sequence element from 3′ UTR
Δ3′DVG: DVG with a sequence element from 5′ UTR
Δ5′3′DVG: DVG lacking sequence elements from 3′ UTR and 5′ UTR
