# Supplementary material for: Biological characterization of coronavirus noncanonical transcripts in vitro and in vivo
Source: Virol J. 2023 Oct 12;20:232. doi: 10.1186/s12985-023-02201-0 (PMC10571414; doi:10.1186/s12985-023-02201-0)
Supplement: Supplementary file 1 — Additional file 1 of Biological characterization of coronavirus noncanonical transcripts in vitro and in vivo. [file 12985_2023_2201_MOESM1_ESM.docx]

**Supplementary data**

**Biological characterization of coronavirus noncanonical transcripts *in vitro* and *in vivo***

**Ching-Hung Lin^1^, BoJia Chen^2^, Day-Yu Chao****^2^, Feng-Cheng Hsieh^1^, Chien-Chen Lai^3^, Wei-Chen Wang^3^, Cheng-Yu Kuo^3^, Chun-Chun Yang^1^, Hsuan-Wei Hsu^1^, Hon-Man-Herman Tam^1^, Hung-Yi Wu^1*^**

**1 Graduate Institute of Veterinary Pathobiology, College of Veterinary Medicine, National Chung Hsing University, Taichung 40227, Taiwan**

**2 Graduate Institute of Microbiology and Public Health, College of Veterinary Medicine, National Chung Hsing University, Taichung 40227, Taiwan**

**3 Institute of Molecular Biology, College of Life Sciences, National Chung Hsing University, Taichung 40227, Taiwan**

***Hung-Yi Wu** <Tel:886-4-22840369>; Fax:886-4-22862073; Email: [hwu2@dragon.nchu.edu.tw](mailto:hwu2@dragon.nchu.edu.tw)


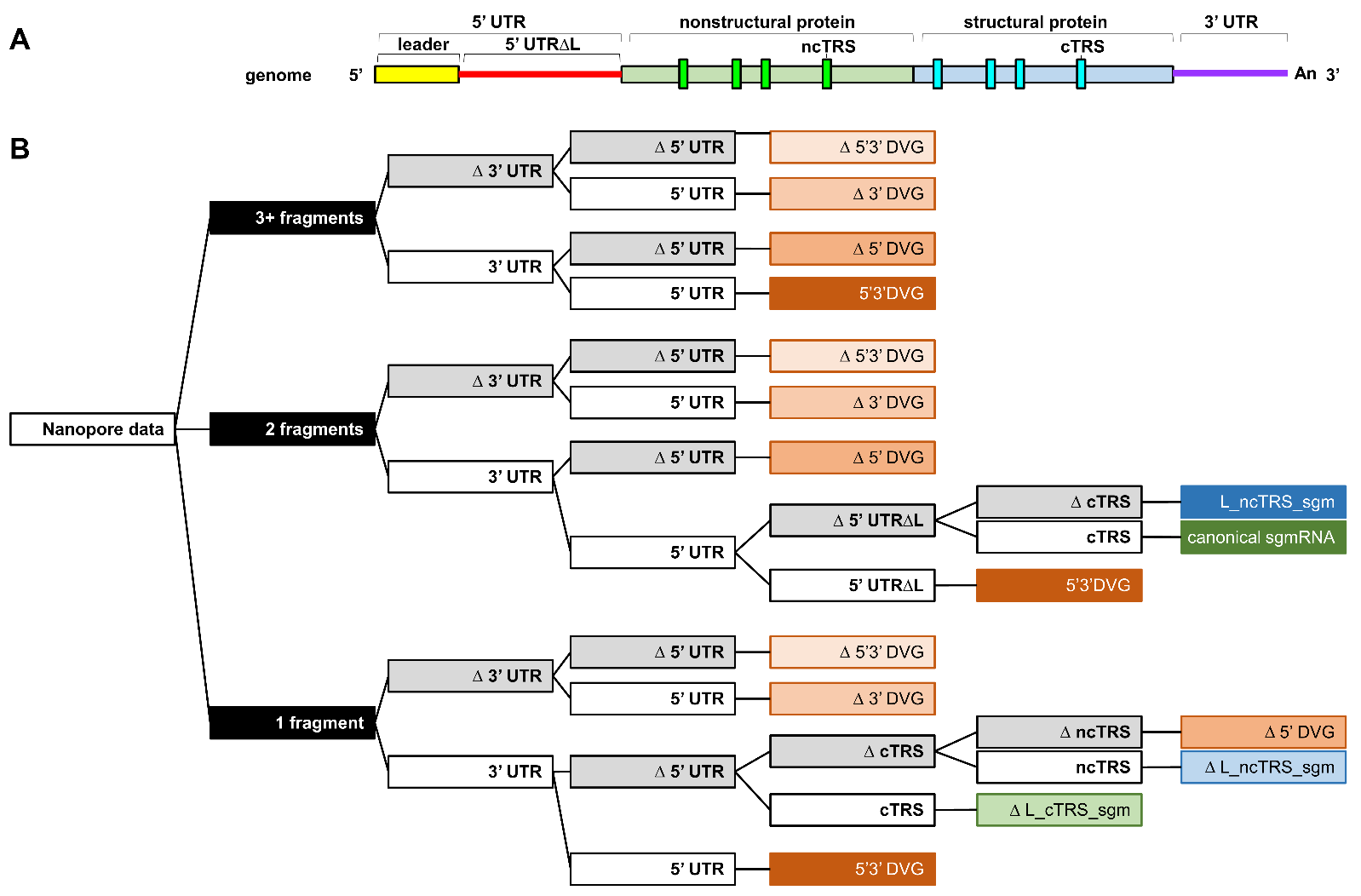
**Figure S1. The method used for the classification of noncanonical transcripts. (A)** Schematic diagram of the coronavirus genome structure. L, leader; 5’ UTRΔL, 5’ UTR without leader sequence; ncTRS, body noncanonical TRS; cTRS, body canonical TRS. **(B)** The flow chart for the classification of noncanonical transcripts. The rationales for the classification are as follows. First, because coronavirus bears the recombination nature, the synthesized transcripts may contain one or more than one fragment of sequences from the coronavirus genome. Thus, TRS-irrelevant transcripts (that is, DVGs) may consists of 1 or more than 1 fragment of sequences from the coronavirus genome. Second, based on the TRS-related sgmRNA synthesis mechanism, sgmRNAs contain the genome body (including 3’ UTR) and the canonical TRS (cTRS) or noncanonical (ncTRS)-derived leader sequence and thus are defined in the classification of 2 fragments. Consequently, the leader-less sgmRNAs contain only genome body, and their 5’ sequences are derived from those positioned within the 50 nucleotides of cTRS or ncTRS. Thus, the leader-less sgmRNAs are defined in the classification of 1 fragment. Based on the criteria defined above, the canonical and noncanonical transcripts are classified in the order of (i) the number of fragments, (ii) with or without 3’ UTR, (iii) with or without 5’ UTR and (iv) whether they are TRS-relevant. Specifically, for the transcripts with more than 2 fragments (that is, 3+ fragments), based on whether the transcripts contain either the partial or complete sequence elements of 3’ UTR and/or 5’ UTR, the transcript species Δ5’3’DVG, Δ3’DVG, Δ5’DVG and 5’3’ DVG are classified. For the transcripts with 2 fragments (that is, 2 fragments) which are irrelevant to TRS, based on whether the transcripts contain either the partial or complete sequence elements from 3’ UTR and/or 5’ UTR, the transcript species Δ5’3’DVG, Δ3’DVG and Δ5’DVG are classified. For the transcripts with 2 fragments and with partial or complete sequence elements from 3’ UTR and 5’ UTR, based on whether transcripts contain partial or complete 5’ UTRΔL (that is, 5’ UTR without leader sequence as shown in (A)) and whether the sequence is relevant to the cTRS or ncTRS, the transcript species are further classified into 5’3’DVG, L_ncTRS_sgm and canonical sgmRNA. For the transcripts with 1 fragment which are irrelevant to TRS, based on whether the transcripts contain either partial or complete sequence elements of 3’ UTR and/or 5’ UTR, the transcript species Δ5’3’DVG, Δ3’DVG and 5’3’DVG are classified. For the transcripts with 1 fragment containing partial or complete sequence elements of 3’ UTR, but no 5’ UTR, based on the whether the 5’ sequences can be derived from those positioned within the 50 nucleotides of cTRS or ncTRS, the transcript species are further classified into Δ5’DVG, ΔL_ncTRS_sgm and ΔL_cTRS_sgm.


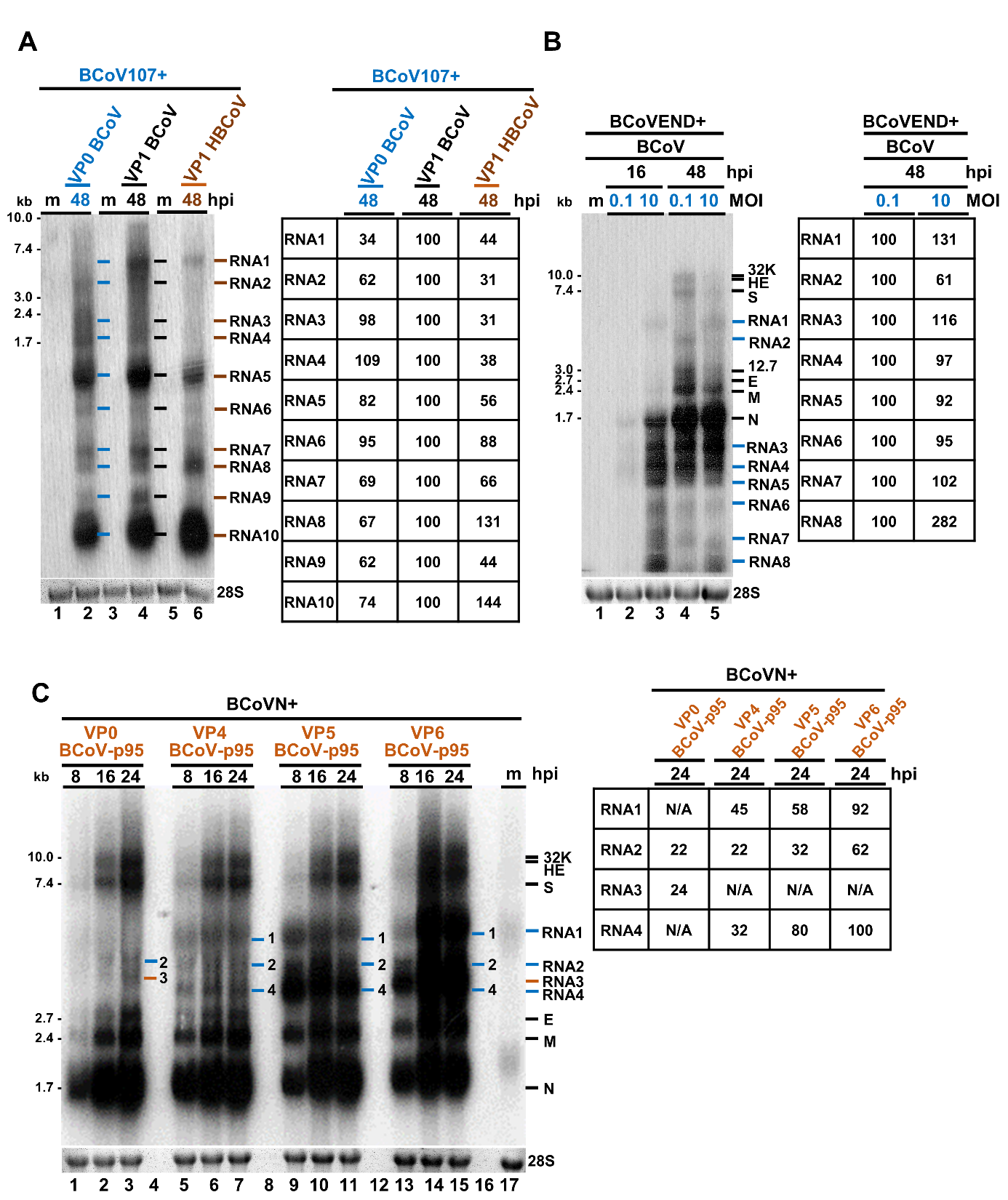


**Figure S2. Quantitation of Northern blotting-detected noncanonical transcripts (shown in Figure 4) synthesized from cells infected with different passages or origins of BCoV (A), different MOI of BCoV (B) or BCoV variant BCoV-p95 (C).** Values in the right panels of figures (A), (B) and (C) represent the percentage of relative quantitation between RNA species detected by Northern blotting. kb, kilobase; m, mock-infected cells; hpi, hours postinfection: 28S, 28S rRNA; VP, viral passage; N/A, not available.

**Table S1. The number of reads for each transcript from mouse liver and ML cells**

**infected with MHV-A59**

|  | MHV-liver | | MHV-cell | |
| --- | --- | --- | --- | --- |
|  | RNA1 | RNA2 | RNA1 | RNA2 |
| Transcripts | Reads | Reads | Reads | Reads |
| canonical sgmRNA | 86 | 40 | 124441 | 51109 |
| ∆L_cTRS_sgm | 28 | 23 | 14718 | 5212 |
| L_ncTRS_sgm | 1 | 0 | 817 | 290 |
| ∆L_ncTRS_sgm | 148 | 111 | 31140 | 9010 |
| 5'3'DVG | 31 | 15 | 36548 | 14930 |
| ∆3'DVG | 9 | 1 | 5441 | 2952 |
| ∆5'DVG | 2887 | 1221 | 306616 | 139522 |
| ∆5'3'DVG | 35 | 17 | 6538 | 3500 |
| Others | 89 | 44 | 5622 | 2318 |
| MHV-A59 RNA | 3314 | 1472 | 531881 | 228843 |

ΔL_cTRS_sgm, leader-less sgmRNA derived from canonical TRS; L_ncTRS_sgm,

sgmRNA derived from noncanonical TRS; ΔL_ncTRS_sgm, leader-less sgmRNA derived

from noncanonical TRS; 5’3’DVG, DVG with sequence elements from 3’ UTR and 5’ UTR;

Δ5’DVG, DVG with a sequence element from 3’ UTR; Δ3’DVG, DVG with a sequence

element from 5’ UTR; Δ5’3’DVG, DVG lacking sequence elements from 3’ UTR and 5’ UTR.

**Table S2. Primers used for Northern blotting assay**

| primers | sequences (5' to 3') |
| --- | --- |
| BCoV 107 (+) | ccactatgaaaaatctacgccc |
| BCoV N (+) | ccagaacgatttccaaaggacgctct |
| BCoV 19304 (+) | gcaagtggcggatttcaaaggtac |
| BCoV END (+) | gtgattcttccaattggcc |
